# Enhancing the signal-to-noise ratio and generating contrast for cryo-EM images with convolutional neural networks

**DOI:** 10.1107/S2052252520013184

**Published:** 2020-10-24

**Authors:** Eugene Palovcak, Daniel Asarnow, Melody G. Campbell, Zanlin Yu, Yifan Cheng

**Affiliations:** a Department of Biochemistry and Biophysics, University of California San Francisco, San Francisco, CA 94143, USA; b Howard Hughes Medical Institute, University of California San Francisco, San Francisco, CA 94132, USA

**Keywords:** cryo-EM, convolutional neural networks, signal-to-noise ratio, contrast

## Abstract

In cryogenic electron microscopy (cryo-EM) of radiation-sensitive biological samples, both the signal-to-noise ratio (SNR) and the contrast of images are critically important in the image-processing pipeline. Classic methods improve low-frequency image contrast experimentally, by imaging with high defocus, or computationally, by applying various types of low-pass filter. These contrast improvements typically come at the expense of the high-frequency SNR, which is suppressed by high-defocus imaging and removed by low-pass filtration. Recently, convolutional neural networks (CNNs) trained to denoise cryo-EM images have produced impressive gains in image contrast, but it is not clear how these algorithms affect the information content of the image. Here, a denoising CNN for cryo-EM images was implemented and a quantitative evaluation of SNR enhancement, induced bias and the effects of denoising on image processing and three-dimensional reconstructions was performed. The study suggests that besides improving the visual contrast of cryo-EM images, the enhanced SNR of denoised images may be used in other parts of the image-processing pipeline, such as classification and 3D alignment. These results lay the groundwork for the use of denoising CNNs in the cryo-EM image-processing pipeline beyond particle picking.

## Abstract 2

It is demonstrated that a convolutional neural network denoising algorithm can be used to significantly enhance the signal-to-noise ratio and generate contrast in cryo-EM images. It also provides a quantitative evaluation of the bias introduced by the denoising procedure and its influence on image processing and three-dimensional reconstructions.

## 1. Introduction   

In single-particle cryogenic electron microscopy (cryo-EM), high-resolution three-dimensional (3D) structures of bio­logical macromolecules are determined by iteratively aligning and averaging a large number of noisy two-dimensional (2D) projection images of molecules embedded in a thin layer of vitreous ice (Cheng, 2015 ▸). This process requires the identification of individual particles in bright-field images (particle picking), sorting the particle images according to conformational state (classification), iteratively determining the orientation of each particle (alignment) and finally calculating a 3D reconstruction. The success of each of these steps is critically dependent on the signal-to-noise ratio (SNR) of the cryo-EM images at all frequencies (Jensen, 2001 ▸). The frequency-dependent SNR is mathematically well defined (Bershad & Rockmore, 1974 ▸; Frank & Al-Ali, 1975 ▸). The visual contrast of cryo-EM images is less strictly defined but is closely related to the low-frequency amplitudes. Both are fundamentally limited by the radiation sensitivity and transparency of the frozen-hydrated cryo-EM specimen, which necessitates low-dose phase-contrast imaging (Glaeser, 1999 ▸). They are further limited by the shape of the contrast transfer function (CTF), which is a sine function and suppresses the amplitude at the low-spatial frequencies that are responsible for producing image contrast (Wade, 1992 ▸).

Within these constraints, the SNR of cryo-EM images is typically well below 1 (more noise than signal; Frank & Al-Ali, 1975 ▸). The conventional approach to maximizing low-frequency SNR and increasing image contrast is to increase the defocus of the objective lens. Higher defocus alters the CTF of the electron microscope, producing higher amplitudes and thus a greater contrast and SNR at low spatial frequencies. This comes at the expense of reducing the amplitude (and thus the SNR) at the intermediate and high spatial frequencies required for high-resolution structure determination (Cheng, 2015 ▸). This intrinsic issue becomes more acute for small or irregularly shaped particles and greatly increases the difficulty of determining the 3D structures of such specimens (Herzik *et al.*, 2019 ▸).

Another method to generate image contrast is to use a phase plate to induce a phase shift in the CTF, improving the contrast at low spatial frequencies without intentionally perturbing the information at high spatial frequencies (Danev & Nagayama, 2001 ▸). The most successful phase-plate device currently available is the Volta phase plate, which is a continuous carbon film placed in the back focal plane (Danev *et al.*, 2017 ▸). In practice, however, the Volta phase plate does in fact cause a noticeable loss of SNR at high frequency, although the precise reason remains unknown (Buijsse *et al.*, 2020 ▸). A new type of laser phase plate that is currently being developed will presumably not have such issues (Schwartz *et al.*, 2018 ▸, 2019 ▸). Nevertheless, complementary and alternative methods of image-contrast enhancement in cryo-EM could therefore be of great value.

Here, we evaluate the performance of a novel computational image-restoration approach that has recently been demonstrated by several groups to increase contrast in cryo-EM images (Buchholz *et al.*, 2019 ▸; Tegunov & Cramer, 2019 ▸; Bepler *et al.*, 2019 ▸). The basic idea of this approach is to train a parameterized image operator (a convolutional neural network; CNN) as an image denoiser. The training scheme used in our approach and others, called *noise*2*noise* (Lehtinen *et al.*, 2018 ▸), uses noisy cryo-EM data as a training signal and is fully compatible with existing strategies for cryo-EM data acquisition. As similarly demonstrated previously by others, we show that CNNs trained with *noise*2*noise* significantly enhance the contrast of cryo-EM images, similar to the effects of the Volta phase plate. We further show that in terms of SNR, the denoising CNNs greatly reduce the noise power at all spatial frequencies. At low and intermediate spatial frequencies, this corresponds to a genuine increase in the relative strength of the true signal in the image. At higher spatial frequencies, noise reduction by CNNs also introduces false signal or ‘bias’. We developed general methods to quantify the bias induced by the denoiser and evaluated its influence on image alignment and 3D reconstruction. Bias introduced by denoising CNNs does not prevent the highly accurate alignment of denoised particles and largely averages out during 3D reconstructions. It is likely that the practical and broad use of denoised images in all stages of the cryo-EM image-processing pipeline will require some minor adaptations of the existing 3D reconstruction software to account for the heavily modulated amplitude spectra. However, our results demonstrate that there is no reason, in principle, why denoising CNNs could not be used to great benefit in single-particle cryo-EM. Our quantitative characterization of the influence of denoising CNNs on the SNR broadens the potential applications of denoising CNNs in single-particle cryo-EM image-processing pipelines.

## 2. Training a convolutional neural network to denoise cryo-EM images   

CNNs are powerful parameterized function approximators (Krizhevsky *et al.*, 2017 ▸). They consist of a large number of small convolution filters with learnable parameters. Each convolutional filter is applied to the input image in real space and passed through a pixel-wise nonlinear function with a shape parameter (typically a threshold-based masking function called a ‘rectified linear unit’ or ‘ReLU’). By stacking a large number of these simple operations in series (convolutional layers), complex input–output maps (functions) can be approximated (Goodfellow *et al.*, 2016 ▸). Since each operation in a CNN is differentiable, the parameters of a CNN can be learned from a large set of input–output image pairs using gradient-based stochastic optimization. To train a CNN to approximate an image denoiser, these input–output pairs typically consist of an image with and without noise (Zhang *et al.*, 2017 ▸). Training consists of calibrating the parameters of the CNN such that applying the CNN to the noisy image produces the noiseless image. To ensure that the CNN generalizes properly to unseen images, the content of the images and the distribution of the noise in the training data set must be representative of those expected during use. These requirements, however, pose a major problem for training a denoising CNN for cryo-EM images: the radiation sensitivity of frozen-hydrated biological samples makes it impossible to obtain a noiseless cryo-EM image in principle (Glaeser, 1999 ▸).

Lehtinen and coworkers recently demonstrated that pairs of noisy images of the same object can be used in place of noisy/noiseless pairs to train denoising CNNs (Lehtinen *et al.*, 2018 ▸). Training is performed as if the noisy image pair were a noisy/noiseless pair: the CNN is applied to the first noisy image and the discrepancy between the output and the second noisy image is calculated (the loss). The loss is used to calculate the direction in which each parameter should be optimized to make the output and the second noisy image more similar (the loss gradient). Lehtinen and coworkers showed that given a sufficiently large set of noisy image pairs, the parameters of the denoiser converge on the same parameter values as would be obtained using conventional noisy/noiseless training data.

This training strategy, termed *noise*2*noise*, enables the parameters of a denoising CNN to be learned without ever seeing noiseless images. It simply requires that the noise in the training pairs be statistically uncorrelated given the signal. In fact, *noise*2*noise* training makes no other assumptions about the structure of the signal or the distribution of the noise and learns both implicitly from the training data. A more mathematical description of the *noise*2*noise* training scheme is provided in Section S1, and we also refer the reader to the original *noise*2*noise* paper and the more general framework for self-supervised denoising described in *noise*2*self* (Batson & Royer, 2019 ▸; Lehtinen *et al.*, 2018 ▸).

Nowadays, cryo-EM images recorded using direct electron-detection cameras are dose-fractionated movies containing many frames. We can generate two images with the same signal but uncorrelated noise by summing up disjoint sets of movie frames, such as the even and odd frames after motion correction (Zheng *et al.*, 2017 ▸), and electron dose-weighting (Grant & Grigorieff, 2015 ▸). Applying the *noise*2*noise* scheme to cryo-EM images therefore requires no alteration in data collection and can work on previously collected data sets.

Note that the noise considered here is mostly shot noise and not amorphous features in the sample such as particle debris or the background added by vitreous ice. Such features are considered ‘structural noise’ when calculating 3D reconstructions, but the denoising CNN has no way of distinguishing them from the desired particle signal and cannot remove them. Nevertheless, shot noise is the dominant source of noise in cryo-EM imaging (Baxter *et al.*, 2009 ▸).

## 3. Results   

### 3.1. Implementing a denoising CNN for cryo-EM   

In principle, the *noise*2*noise* training scheme only requires a large data set of noisy image pairs to train a denoising CNN. In practice, we found that the denoising performance depends strongly on the structure of the CNN and the preprocessing of the training images. We implemented the *noise*2*noise* procedure in a denoising program, *restore*, in which we used a CNN architecture similar to the U-net used by Lehtinen and coworkers but with some modifications (Ronneberger *et al.*, 2015 ▸). We replaced each block of convolutional layers in the U-net with a wide-activation convolutional layer (Yu *et al.*, 2018 ▸) and used depth-to-space up-sampling to minimize aliasing artifacts (Odena *et al.*, 2016 ▸). We found that these modifications improved the training loss and the visual quality of the output compared with a standard U-net. In the supporting information, we provide a full description of the CNN architecture used in this work (Supplementary Fig. S2, Section S1).

Currently, for each specific cryo-EM data set, we use part of the data set as training pairs to train a specific CNN and use the trained CNN to denoise cryo-EM images of the entire data set. This is possible because a typical cryo-EM data set consists of thousands of movies and the training process is relatively fast (a few hours). With such an abundance of training data, overfitting the CNN is unlikely.

To generate training data from a set of dose-fractionated electron movies, we first generate motion-corrected and dose-weighted sums of the even and odd frames using *MotionCor*2 (Zheng *et al.*, 2017 ▸). We have tested our procedure on a number of examples (discussed below). The physical pixel sizes of these data sets are approximately 1 Å. Each cryo-EM image is modified by the CTF of the electron microscope. Considering that the defocus and astigmatism of each image is different, the CTF modifies otherwise similar signals in unique but predictable ways, and a denoising CNN would need to learn to distinguish all CTF-aberrated signals from noise. We reasoned that it would be advantageous to correct the CTF in the noisy images upfront, so that the CNN would not need to learn to be invariant to CTF modulations of the underlying signal. Thus, before training and applying the CNN to denoise all images in a data set, we performed phase-flipping on the images by multiplying their Fourier transforms by the sign of the CTF and computing the inverse Fourier transform. While the examples we show in the following are phase-flipped prior to training the CNN and denoising the image, it may be unnecessary to phase-flip the training images prior to the denoising procedure.

Considering that the spectral SNR at high spatial frequencies is very low [*i.e.* below ∼0.25 beyond 0.2 Å^−1^ in Fig. 2(*e*)] (Baxter *et al.*, 2009 ▸), we do not expect any denoising algorithm to recover the signal under such conditions. We typically bin training images by Fourier cropping, which effectively reduces the total noise in the image and makes it smaller. In the example of the 20S proteasome shown later, we Fourier crop the training images to a pixel size of ∼1.5 Å. To facilitate training in batches, we break up the phase-flipped, Fourier-cropped training images into square patches (typically 192 × 192 pixels). For most of the macromolecular specimens examined, a patch of this size contains at least several particles. We normalize each patch by subtracting the mean pixel value and dividing by the standard deviation.

Finally, we train the CNN using the *Adam* stochastic optimization algorithm with weight normalization (Kingma & Ba, 2014 ▸; Salimans & Kingma, 2016 ▸). We find that training CNNs with *noise*2*noise* is fast, typically achieving a stable plateau in the value of the loss function in several epochs (20–30 min on a single modern GPU). We train for 100 epochs.

Once trained, the CNN can be used to denoise full images. We preprocess these images in the same way as during training, except that we do not break the image into patches. This is possible because CNNs can operate on images with arbitrary shape and size. Finally, we unbin the denoised image by zero-padding its Fourier transform, low-pass filtering with a soft radial Fourier mask and calculating the inverse Fourier transform. The result is a denoised image with the shape and pixel size of the original image, band-limited to the same frequency as the binned training data.

### 3.2. Contrast enhancement for diverse cryo-EM specimens   

**Figure 1:**
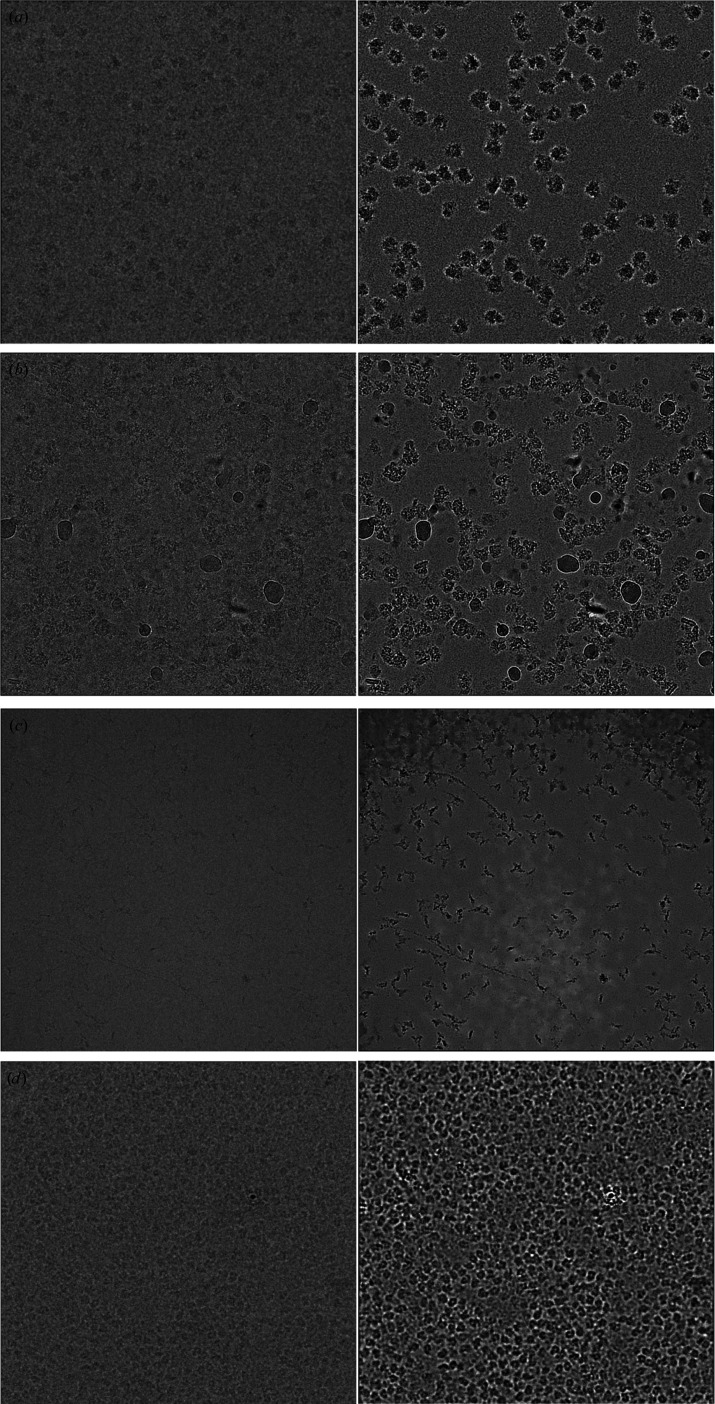
Performance of denoising CNNs on real cryo-EM images. (*a*) *P. falciparum* ribosome particles, (*b*) TRPM4 particles, (*c*) integrin–Fab particles and (*d*) protein kinase A particles before (left) and after (right) denoising.

We trained denoising CNNs on several previously collected cryo-EM data sets with a range of specimen types and imaging conditions: the *Plasmodium falciparum* 80S ribosome (∼3.2 MDa), the human TRPM4 ion channel (∼700 kDa), a human integrin–Fab complex (∼280 kDa) and the catalytic domain of human protein kinase A (∼43 kDa). Despite differences in particle shape, molecular weight and imaging conditions, all of the denoised images show significantly enhanced visual contrast, with particle shapes that are clearly defined and consistent with the shape of these molecules [Figs. 1 ▸(*a*)–1 ▸(*d*)] (Autzen *et al.*, 2018 ▸; Campbell *et al.*, 2020 ▸; Herzik *et al.*, 2019 ▸; Wong *et al.*, 2014 ▸). We show four cases here, but we have not found any case in which the contrast of single-particle cryo-EM specimens is not significantly enhanced by training and applying a denoising CNN.

**Figure 2:**
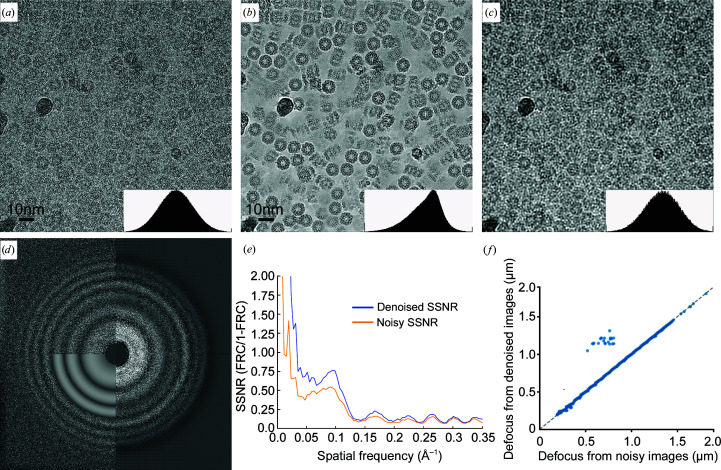
Effects of denoising on Fourier amplitudes. (*a*) Cryo-EM image of 20S proteasome recorded at a defocus of 0.4 µm. (*b*) The same cryo-EM image after denoising. (*c*) The same image after applying a low-pass filter in Fourier space to 1/20 Å. The image contrast in (*a*), (*b*) and (*c*) is manually scaled so that the histograms of pixel intensities are similar (small insert in each panel). (*d*) Fourier transforms calculated from the original (upper left) and denoised cryo-EM images (right). Thon-ring simulation for CTF determination is shown on the lower left. (*e*) Spectra signal-to-noise ratio (SSNR) profile calculated from cryo-EM images before (orange) and after (blue) denoising. SSNR = FRC/(1 − FRC), where FRC is calculated between sums of even and odd frames. (*f*) Scatter plot of defocus values determined from images before and after denoising. Defocus values were estimated using *Gctf* (Zhang, 2016 ▸) and the major and minor defocus values were averaged. The small population of off-diagonal images (24 of 843) appear to be heavily contaminated with crystalline ice.

To understand how a denoising CNN affects information at different spatial frequencies in the image, we trained a denoising CNN on a data set for a well characterized test specimen: the *Thermoplasma acidophilum* 20S proteasome (T20S; Fig. 2 ▸). For this data set, we used all images in the data set to train the denoiser and we band-limited the resolution of the training data to 3 Å by Fourier cropping. Compared with the original image [Fig. 2 ▸(*a*)], the denoised image [Fig. 2 ▸(*b*)] shows significant image contrast without the blurring shown in the low-pass-filtered image [Fig. 2 ▸(*c*)]. The Fourier power spectrum [Fig. 2 ▸(*d*)] and spectral SNR (SSNR) [Fig. 2 ▸(*e*)] calculated from the image before and after denoising show that the SNR is boosted at the low-frequency Fourier components without a reduction at high frequency. This behavior is different from a linear Fourier filter (such as the typically used low-pass filter), which boosts the low-frequency amplitude by suppressing the high-frequency amplitude but without any improvement in the SSNR at any frequency. Thon rings associated with the CTF are present and correctly located in the denoised images, and the defocus values estimated from the denoised images are mostly close to those estimated from the original noisy image [Figs. 2 ▸(*d*) and 2 ▸(*f*)]. A few images that have obviously different estimated defocus values after denoising [upper left quadrant of Fig. 2 ▸(*f*)] are heavily contaminated with crystalline ice.

These examples demonstrate that CNNs trained by the *noise*2*noise* scheme are effective in denoising and contrast enhancement on a wide range of real single-particle cryo-EM specimens. Visualization of particles is significantly enhanced, facilitating more efficient manual image evaluation and particle picking. While we preprocess our images differently, use a novel CNN architecture and train a single CNN model per data set, our results are broadly consistent with other efforts using similar denoising approaches (Bepler, Morin *et al.*, 2019 ▸; Bepler, Noble *et al.*, 2019 ▸; Tegunov & Cramer, 2019 ▸) and illustrate the robustness of the *noise*2*noise* algorithm.

### 3.3. Quantitative estimation of signal enhancement and noise suppression   

A major question concerning the denoising procedure that we describe here is whether the signal is faithfully retained at all spatial frequencies. A related question is whether the denoising procedure can facilitate any other steps in the single-particle cryo-EM pipeline beyond the visual evaluation of images and particle picking.

To answer these questions, we extended the conventional cross-correlation-based estimators for SNR and spectral SNR (SSNR) to handle images modified by a deterministic, arbitrary operation such as denoising (Section S3; Baxter *et al.*, 2009 ▸; Bershad & Rockmore, 1974 ▸; Frank & Al-Ali, 1975 ▸). Our approach estimates the magnitude of any ‘false signal’ or ‘bias’ added to each image during denoising. This is possible because pairs of denoised images of the same object will share signal and bias in common, while a denoised image and its paired noisy image will only share the signal. By estimating the signal variance, bias variance and noise variance, we can compute quantities such as SNR and other similar quantities that take bias into account. Importantly, we can compute these quantities as a function of spatial frequency.

**Figure 3:**
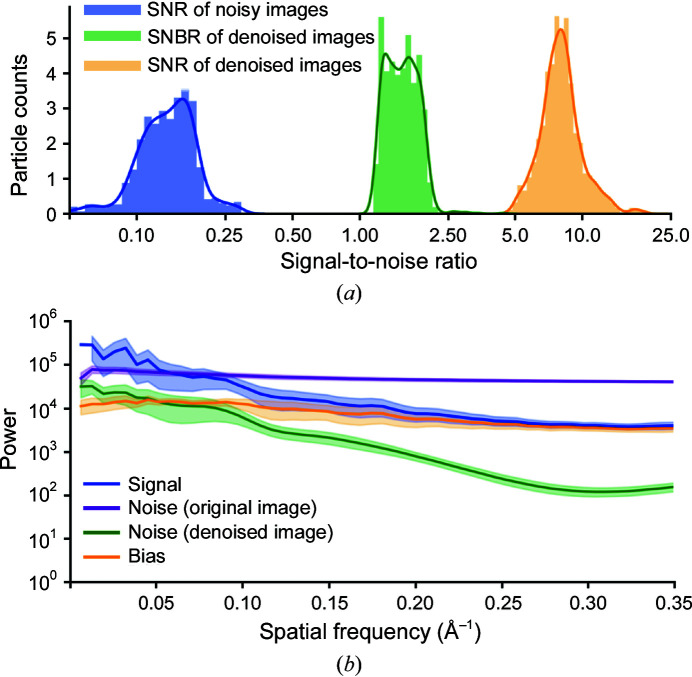
Quantitative analysis of signal, noise and bias in denoised images. (*a*) Histograms of SNRs before (blue) and after (orange) denoising and SNBRs after denoising (green) for images from the T20S proteasome data set. Smooth lines represent kernel density estimates of the distribution. The *x* axis is on a logarithmic scale. (*b*) Spatial frequency-dependent variance (power) of the signal (blue), bias (orange) and noise. Noise power is calculated before (purple) and after (green) denoising. The *y* axis is on a logarithmic scale. Curves represent the mean of the quantities for all images in the T20S proteasome data set. Shaded regions show one standard deviation above and below each mean curve. All quantities were calculated as described in Sections S2 and S3.

We estimated the SNR for noisy and denoised images of the entire T20S data set [Fig. 3 ▸(*a*)]. While the mean SNR of the noisy images is 0.14, the mean SNR of the denoised images is 8.3. This enhancement, however, could have resulted from some noise being transformed into bias by the denoiser, inducing spurious correlations between denoised images. We define another quantity, the signal-to-noise-and-bias ratio (SNBR), which is the ratio of the signal variance and the sum of the noise and bias variances (Section S3). Intuitively, the SNBR represents the relative power of true signal compared with the power of all other components in the image. For the T20S data set the mean SNBR is 1.4, which is still a significant improvement over the original SNR of the noisy images.

We estimated the frequency-dependent variance (power) of the signal, bias and noise before and after denoising [Fig. 3 ▸(*b*)]. When we plot the average of these quantities over all micrographs in the T20S data set, we find that the noise in the original image dominates the signal at all but the lowest spatial frequencies. After denoising, the noise is much smaller than the signal at all spatial frequencies. Additionally, the signal is significantly larger than the bias at low spatial frequencies (>0.1 Å^−1^) but has similar power at higher spatial frequencies.

Taken together, these results indicate that denoising increases the strength of the true signal in the denoised images by a sizable factor. However, denoising also transforms a portion of the uncorrelated noise into statistically correlated bias, which is undesirable. The nature of this bias is not clear. One possible interpretation is that high spatial frequency patterns of signal may be impossible to disambiguate in images with low SNR. For example, the precise arrangement of side chains in an image of a folded protein will be encoded by high spatial frequency Fourier components. When heavily corrupted with noise, an image of such a signal may have several plausible interpretations. Because the CNN could be wrong about any particular arrangement of side chains, the best guess for minimizing the mean-squared error is a pixel-wise average over all possible interpretations. This average would appear blurred and would match no single arrangement exactly, but would not be far off (in the mean-squared error sense) from any single plausible arrangement.

If this is the case, low spatial frequencies should be relatively less biased than high spatial frequencies in denoised images because the higher SNR at low spatial frequencies should make it easier for the CNN to identify the signal unambiguously. This is what we observe in Fig. 3 ▸(*b*) and is consistent with the denoising CNN enhancing the true signal at low spatial frequencies while mostly transforming the noise into bias at high spatial frequencies.

### 3.4. 3D reconstructions with denoised particles   

There are two questions concerning the bias being introduced into the denoised images. Firstly, is the bias correlated between images of different particles? Assuming that the correct angular orientation of each individual particle image is known, correlated bias will generate artificial structural features in the 3D reconstruction, but uncorrelated bias will be averaged out without generating artifacts. Secondly, would such bias interfere with the image-alignment procedure and prevent direct structure determination from denoised images? We explore these two questions by performing 3D reconstructions and refinements on denoised images of the 20S proteasome.

**Figure 4:**
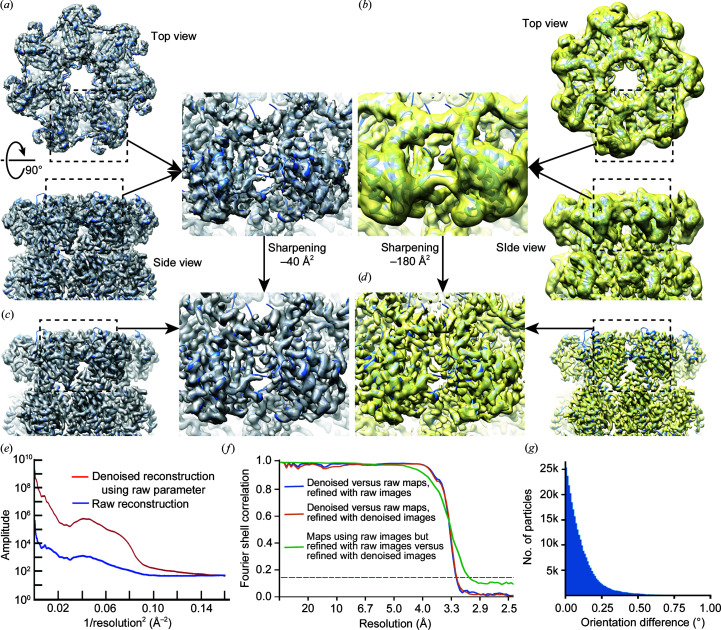
3D reconstructions of denoised T20S proteasome images. (*a*) Reconstruction of the original particle images without sharpening for the T20S proteasome in top (upper) and side (bottom) views. Iterative structure determination and refinement were performed using *cryoSPARC* (Punjani *et al.*, 2017 ▸) with *D*7 symmetry. For consistency with the other panels, the final reconstruction was calculated by transferring all parameters into *RELION* and using *relion_reconstruct* without symmetry (Zivanov *et al.*, 2018 ▸). (*b*) Reconstruction of denoised particle images in top (upper) and side (bottom) views, with the same orientation parameters as used in (*a*). (*c*) Reconstruction of the original particles after sharpening by −40 Å^2^. (*d*) Reconstruction of the denoised particles after sharpening by −180 Å^2^. (*e*) Comparison of rotational averages of the Fourier amplitude of reconstructions of original images (blue) and denoised images (red) calculated using the same parameter refined from the original images. (*f*) FSC curves between reconstructions of original and denoised particles using orientational parameters determined from the original particles (blue), between reconstructions of original and denoised particles using parameters determined from the denoised particles (orange) and between reconstructions of original particles using parameters determined from either the original or denoised particles (green). (*g*) Histogram of errors in the orientation parameters estimated during the refinement of denoised particles.

Using the T20S proteasome data set mentioned above, we performed standard single-particle cryo-EM structure determination on the original noisy micrographs, from particle picking to iterative refinement and 3D reconstruction, using an initial model that was calculated from the atomic model of T20S proteasome and low-pass filtered to 60 Å. The final reconstruction was refined to 3.3 Å resolution [estimated from the Fourier shell correlation (FSC) = 0.143 criterion (Rosenthal & Henderson, 2003 ▸)] from 302 290 particles using *cryoSPARC* without applying symmetry [Fig. 4 ▸(*a*), Supplementary Fig. S2(*a*), blue curve]. Even without sharpening, densities for side chains are clearly visible. This reconstruction and the orientational parameters of each particle in the final data set are then treated as references to quantitatively evaluate the behavior of denoised particles in both 3D reconstruction and structure refinement.

We extracted the same particles from the denoised micrographs and calculated a 3D reconstruction using *relion_reconstruct* by using the orientation and CTF parameters determined from the original noisy images [Fig. 4 ▸(*b*)]. The reconstruction of denoised particles has the correct overall shape and some detailed structural features, but appears to be significantly blurred compared with the 3D reconstruction of the original particles [Fig. 4 ▸(*b*)], although the resolution estimation from FSC extends to 3.4 Å [Supplementary Fig. S2(*b*), red curve]. The resolution is not uniform, as some helices are well resolved, with visible helical grooves, while others are completely unresolved [Fig. 4 ▸(*b*)]. However, after sharpening by a negative *B* factor, −40 Å^2^ for the raw particle reconstruction and −180 Å^2^ for the denoised particle reconstruction, all high-resolution features, including side-chain densities, are similar in both reconstructions [Figs. 4 ▸(*c*) and 4 ▸(*d*)]. The rotational averaged Fourier amplitudes of these two density maps also indicate the need for a larger negative *B*-factor sharpening for the reconstruction of denoised images [Fig. 4 ▸(*e*)]. An FSC curve calculated from the two reconstructions is close to 1 before 4 Å and falls off at 3.3 Å [Fig. 4 ▸(*f*), blue curve]. Because the denoised particles are band-limited to 1/3 Å^−1^, this suggests that the denoised images contain high-resolution information until nearly the point where it was explicitly truncated. Importantly, it also suggests that the bias introduced by the denoising procedure is sufficiently random and can be removed by averaging large numbers of particle images. The large *B *factor needed to sharpen the reconstruction of denoised particles may be caused by the significantly enhanced low-frequency SNR of denoised particle images.

We further used the same stack of denoised particles for a standard iterative procedure of particle alignment and 3D reconstruction using the same initial reference model and *RELION* 3D refinement (*cryoSPARC* does not support the refinement of phase-flipped particle images). The resolution of the final reconstruction estimated by the gold-standard FSC is 3.4 Å [Supplementary Fig. S2(*b*), orange curve]. Similarly, the reconstruction without sharpening shows strong low-resolution features, but *B*-factor sharpening reveals correct high-resolution features [Supplementary Figs. S2(*c*), S2(*d*) and S2(*e*)]. The angular differences in the orientations determined from the original noisy and denoised particles are small [Fig. 4 ▸(*g*)], and the ResLog plots (Stagg *et al.*, 2014 ▸) of both reconstructions are comparable [Supplementary Fig. S2(*f*)]. A 3D reconstruction calculated from original noisy particles but using orientation parameters determined from the denoised particles has a slightly better resolution [3.3 Å; Supplementary Fig. S2(*b*), green curve] and is highly correlated with the 3D reconstruction determined from the original particle images [Fig. 4 ▸(*f*), green curve]. We speculate that the small angular errors are caused either by bias introduced into the denoised images or by the overweighting of low-frequency information during alignment of the denoised images.

## 4. Conclusions and discussion   

Denoising CNNs can significantly enhance the contrast of noisy cryo-EM images. The most immediate applications should be the visual evaluation of specimens before large-scale data collection and particle picking for small or irregularly shaped macromolecules. Beyond these applications, the quantitative evaluation of frequency-dependent signal, noise and bias show that the true signal in the denoised images is enhanced at the low and intermediate spatial frequencies required for particle alignment and is maintained at high frequency.

Iterative refinement of denoised particles leads to reconstructions with nearly correct structural features at high resolution, demonstrating the potential of using denoised particles directly for single-particle cryo-EM structure determinations. Small errors in the orientation parameters determined from denoised images could be corrected by substituting and further refining the original noisy images. This reversibility is advantageous for cryo-EM image processing, unlike phase plates, which improve contrast by irreversibly modulating the image.

Although we have not demonstrated it here, CNN denoising also has the potential to facilitate the better identification of small classes of particles that correspond to weakly populated intermediate states of macromolecular machines. This will be especially true if the intermediate states differ in low- or intermediate-resolution structural features, such as the relative positioning of a protein domain. Considering that the bias is more pronounced at high frequency, it may be desirable to merge denoised and original images in Fourier space by combining the low-frequency signal from the denoised image with the high-frequency signal from the original image. We envision that such merged images could be used for the entire single-particle cryo-EM image-processing pipeline. In the supporting information, we discuss a method of merging the original noisy and denoised particle images. The major impediment to directly using denoised images or merged images for alignment and classification appears to be that the current procedures implemented in widely used cryo-EM software are not tuned to handle the heavily modulated amplitude spectra of denoised images. It may also be useful to consider other uses for denoised images. With higher SNR single particles, the particle is clearly delineated from the background. This would make per-particle real-space masking possible for small particles with irregular shapes, eliminating most of the noise surrounding the particle and presumably enhancing alignment. Similarly, it could make previously proposed pseudo-atom approaches for estimating initial models and measuring macromolecular flexibility more tractable (Joubert & Habeck, 2015 ▸).

## 5. Related literature   

The following references are cited in the supporting information for this article: Abadi *et al.* (2016 ▸), Asarnow *et al.* (2019 ▸), Booth *et al.* (2004 ▸) and Mindell & Grigorieff (2003 ▸).

## Supplementary Material

Supplementary Figures and Supporting Information. DOI: 10.1107/S2052252520013184/pw5015sup1.pdf
