# Supplementary material for: Enhancing the signal-to-noise ratio and generating contrast for cryo-EM images with convolutional neural networks
Source: IUCrJ. 2020 Oct 24;7(Pt 6):1142–50. doi: 10.1107/S2052252520013184 (PMC7642784; doi:10.1107/S2052252520013184)
Supplement: Supplementary file 1 [file m-07-01142-sup1.pdf]

IUCrJ

Volume 8 (2021)

Supporting information for article:

Enhancing the signal-to-noise ratio and generating contrast for cryo-EM images with convolutional neural networks

Eugene Palovcak, Daniel Asarnow, Melody G. Campbell, Zanlin Yu and Yifan Cheng

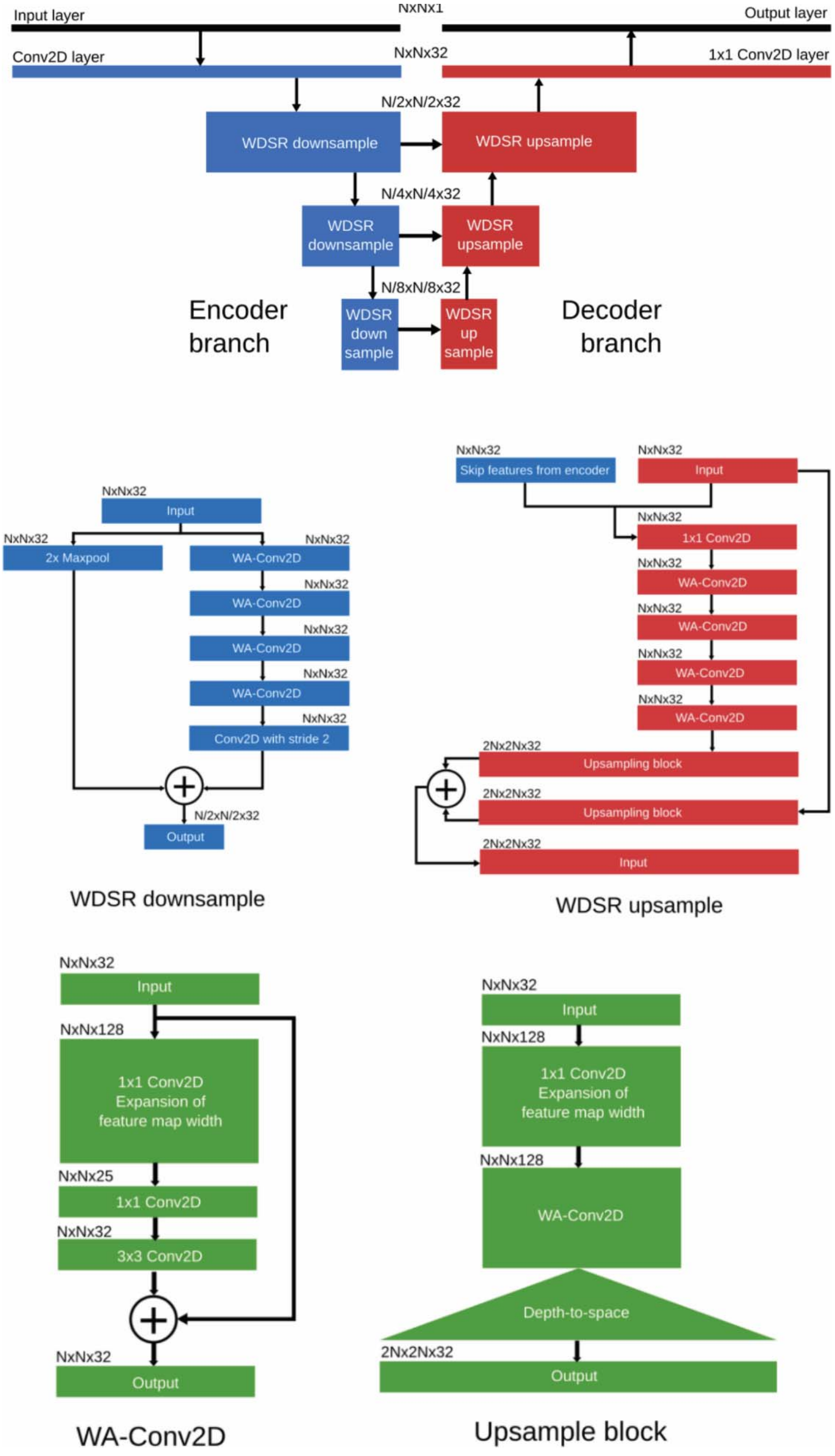
Input layer
NxNx1
Output layer
Conv2D layer
NxNx32
1x1 Conv2D layer
N/2xN/2x32
WDSR downsample
WDSR upsample
N/4xN/4x32
WDSR downsample
WDSR upsample
N/8xN/8x32
Encoder branch
WDSR down sample
WDSR up sample
Decoder branch
NxNx32
Input
2x Maxpool
WA-Conv2D
WA-Conv2D
WA-Conv2D
WA-Conv2D
Conv2D with stride 2
N/2xN/2x32
Output
WDSR downsample
Skip features from encoder
Input
1x1 Conv2D
WA-Conv2D
2Nx2Nx32
Upsampling block
Input
WDSR upsample
NxNx128
1x1 Conv2D Expansion of feature map width
NxNx25
1x1 Conv2D
3x3 Conv2D
Output
WA-Conv2D
Depth-to-space
Upsample block

Figure S1 Architecture of the convolutional neural network**.** (**A**) The network has an encoder-decoder structure, similar to the U-net architectures commonly used in biomedical image segmentation. Each convolutional downsampling or upsampling operation in the CNN is performed by a 'wide-activation super-resolution' (WDSR) sub-network. (**B**) The WDSR down-sample subnetwork (left network) consists of 4 wide-activation convolutional layers (WA-Conv2D, described in C) followed by a conventional convolution with stride 2. The resultant downsampled feature maps are summed with the input feature maps, which are downsampled by 2x max-pooling, as in a residual neural network. The WDSR up-sample network (right) first concatenates the input feature maps and the feature maps from the skip connections and reduces them with a 1x1 convolution operation (NxNx64 → NxNx32). After 4 WA-Conv2D operations, the feature maps are 2x upsampled with an upsampling block (described in C). Similar to the downsampling network, the input feature maps are upsampled and summed with the upsampled output feature maps. (**C**) The WA-Conv2D operation (left) first uses a 1x1 convolution operation to 'expand' the input feature maps into a higher dimensional space (NxNx32-->NxNx128). Intuitively, each feature map of the higher dimensional space is a distinct linear combination of the input feature maps. A ReLU non-linearity is applied to the expanded feature maps, followed by another 1x1 convolution operation to reduce the feature map depth. A conventional convolution operation is then applied with a 3x3 convolutional filter. The upsampling block (right) consists of a WA-Conv2D operation followed by a depth-to-space upsampling operation.

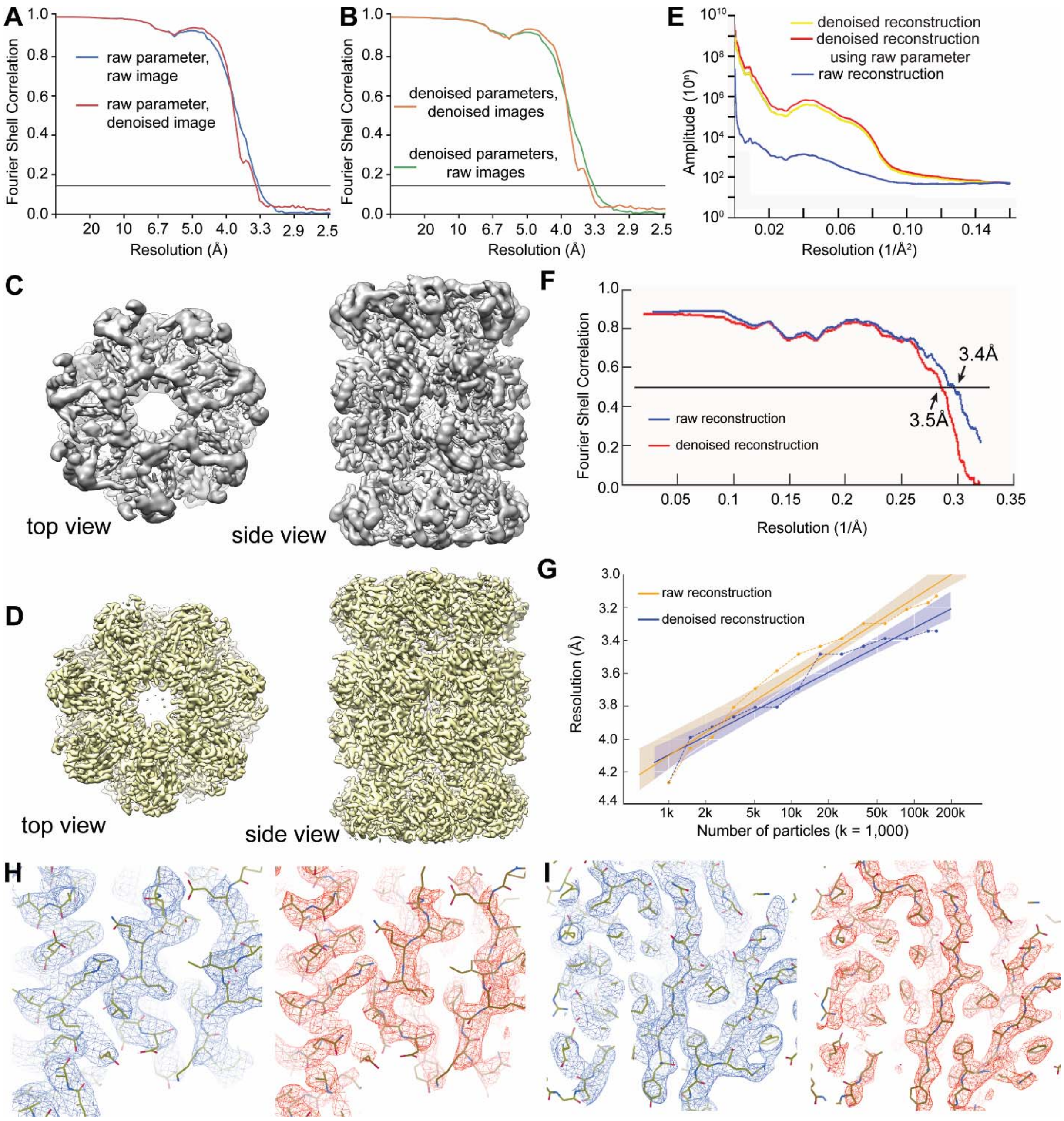

Figure S2 (**A**) Gold standard FSC curve of T20S proteasome determined from original images (blue) and FSC curve of the map reconstructed using the same parameters but denoised particle images (red). (**B**) Gold standard FSC curve of T20S proteasome of denoised images with orientation parameters determined from denoised particle images (orange) and FSC curve of the map reconstructed using the same parameters but with original noisy particle images (green). (**C**) Side and top view of 3D reconstruction determined noisy images, before B-factor sharpening. (**D**) same views of 3D reconstruction sharpened by a B-factor of -180Å^2. (**E**) Comparison of rotational averages of Fourier amplitude of

reconstructions determined from original images (blue), from denoised images using the same parameter refined from original images (red) (the same as blue curve in Figure 2E), and of denoised image refined independently (yellow). (**F**) Overlay of model (PDB code: 3J9I) vs map FSC curves of maps determined separately using raw images (blue) or denoised images (red). (**G**) ResLog plots for the same two reconstructions, using raw (yellow) and denoised images (blue). (**H**) and (**I**) Detailed comparison of side chain densities of two selected regions from the reconstructions determined from raw images (blue) and denoised images (red).

S1. Mathematical description of the *noise*2*noise* training scheme

First, we assume an image (M) to be a vector of pixels composed of a signal vector (S) and an additive noise vector (N). Though we do not explicitly denote it, the noise component may or may not depend statistically on the signal and therefore can include shot noise (Poisson noise).

eq.1 $$M = S + N$$

A parameterized denoiser is a function with parameters θ that takes the noisy image, M, and outputs the signal, S:

eq.2 $$f_\theta(M) = S$$

The conventional strategy for training a deep CNN to be an image denoiser is to search for the set of parameters, θ, that minimizes the expected value of a loss function (the expected risk) over all the clean/noisy image pairs in the training data:

eq.3 $$\text{argmin}_\theta\ E(|f_\theta(M) - S|^p) = \text{argmin}_\theta\ E(|f_\theta(S+N) - S|^p)$$

where E(X) denotes expectation over the data distribution and $|X|^p$ denotes the L_p norm over all pixels in the resulting difference image. In this and other related works, p=2 and corresponds to the mean-squared error over pairs of corresponding pixels, though L_1 (median-seeking) and approximate L_0 (mode-seeking) norms were also demonstrated in Lehtinen et al. These parameters are determined using some variant of stochastic gradient descent over many small batches of the training data. Because f_θ (and any CNN in general) is a differentiable function, the gradient of each parameter with respect to the loss can be estimated directly using the chain rule (the back-propagation algorithm).

The key insight of Lehtinen et al. is that one can learn the same parameters without clean data. Instead of M and S, assume we have two images with the same signal but uncorrelated noise:

eq.4
$$M_1 = S + N_1$$
$$M_2 = S + N_2$$

We make two general assumptions about the noise:

eq.5
$$P(N_2|S,N_1) = P(N_2|S)$$
eq.6
$$E(N_i) = \mathbf{0}$$

where P(N|S) is the conditional probability distribution of the noise given the signal, $E(N_i)$ is the expectation over the distribution of noise, and **0** is a vector of zeros with the same shape as the images M_1 and M_2.. Intuitively, these conditions imply that the noise present in an image pair is statistically independent from each other given the signal and that the noise is zero-mean and ‘averages out' if many noisy images are summed together. Then, we train the CNN with stochastic gradient descent or one of its variants to minimize the following objective:

eq.7
$$\text{argmin}_\theta\ E(\ |f_\theta(M_1) - M_2|^p\ ) = \text{argmin}_\theta\ E(\ |f_\theta(S+N_1) - S - N_2|^p\ )$$

In this procedure, while we are training f_θ to convert one noisy image, M_1, into the second noisy image M_2, because of (eq.5), M_1 provides f_θ with *no information* about the specific instantiation of noise N_2 that it is tasked to predict: N_2 is an independent random draw from the noise distribution P(N|S). To minimize the expected risk, the best guess f_θ can make for N_2 is the average of all the possible instantiations of noise it might see, which by (eq.6) is zero noise. This implies that the objective in (eq.7) is minimized by the same set of parameters **θ** that minimizes (eq.3), and so (eq.7) trains f_θ to be a denoiser.

The *noise2noise* strategy is more intuitive when considered geometrically, when an image is represented by a vector of pixel intensities, locating a single point in a high-dimensional vector space. A signal, S, occupies a point in the space. Noise is a vector N_i that displaces an image away from signal S to a noisy image $S+N_i$. The conventional denoising CNN strategy trains a mapping between a given noisy point $S+N_i$ and S. The *noise2noise* strategy attempts to train a mapping between a given noisy point $S+N_1$ and a second noisy point $S+N_2$, but provides no information about N_2. Because N_2 could be any of the possible

noisy points and we penalize f_θ if its output is distant from $S+N_2$, the least risky guess is the point that is closest to all of the noisy points. This point is simply the mean of all noisy images $S+N_i$, which is also the noiseless signal, S.

S2. Technical description of the training and denoising implementation

Code implementing these methods is available at https://github.com/eugenepalovcak/restore.
All code was written in python. Neural networks were implemented and trained using the libraries keras and Tensorflow (Abadi *et al.*, 2016).

S2.1. I/O and image pre-processing

Our program, *restore*, takes as input a RELION-style STAR file containing the file path and CTF parameters for a set of training micrographs. Each image in this STAR file is assumed to be the sum of all frames summed together after motion correction and dose-weighting with MotionCor2 (Zheng *et al.*, 2017) and CTF estimation with CTFFIND (Mindell & Grigorieff, 2003), Gctf (Zhang, 2016), or some other CTF-estimation program. Each training image is expected to have a training pair of even/odd images. These can be generated with recent versions of MotionCor2 using the '-SplitSum 1' option. In this case, the full sum image has the suffix 'DW' while the even/odd half sum images have the suffixes 'EVN' and 'ODD'.

restore begins by preprocessing the training data. For each even and odd half-sum image, we load the MRC file into an array. Then, we Fourier-crop the micrograph such that the pixel size is ½ the value of the '--max_resolution parameter', effectively setting the nyquist frequency '--max_resolution'. A band-pass filter is applied in Fourier space to remove low frequency information (lower than 1/200 angstroms, corresponding to gradients of image contrast due to changes in ice thickness) and high-frequency information beyond the Nyquist frequency.

Next, we correct for the CTF by phase-flipping. Explicitly, we calculate the amplitude modulation implied by the CTF for each Fourier component, compute the sign of each amplitude modulation (+1 or -1), and multiply the Fourier transform of the Fourier-cropped image by this array. We inverse Fourier transform this image. It is worth noting that we also tried denoising without phase-flipping and found similar results as with phase-flipping. However, we did not extensive test this option.

Then, we break each even and odd half-sum image up into 192x192-pixel patches. We normalize by subtracting the mean and dividing by the standard deviation. We store the patches in a large hierarchical data format (HDF) file. This allows fast access to the preprocessed data during training without requiring the entire dataset to be loaded into memory.

S2.2. CNN architecture

We specified the architecture of the CNN using the keras library. Our CNN has a U-net architecture with specialized CNN block structures. The encoder branch consists of an initial 2D convolutional layer followed by three larger blocks of convolutional layers that progressively down-sample the input. In the decoder branch, three blocks of convolutional layers then progressively upsample the feature maps and receive ‘skip connections’ from the encoder.

The blocks of convolutional layers are modeled after the wide-activation super resolution (WDSR) networks described by Yu et al. The basic building block of these networks is the wide-activation convolutional layer. Wide activation convolutional layers take linear combinations of feature maps to increase their depth before applying a non-linearity and convolution operation. These expanded linear combinations allow more information to pass through the layers without getting decimated by the ReLU non-linearity (the so-called ‘vanishing gradient’ problem). For the same reason, each layer also uses residual connections, adding the processed information to the input.

Wide-activation convolutions are implemented by performing a 1x1 convolution to increase the depth of the input feature maps from 32 to 128, applying a ReLu non-linearity, and performing another 1x1 convolution to reduce the feature maps from 128 to 25. Finally, a 2D convolution is applied with 32 feature maps and 3x3 kernel size. A down-sampling wide-activation block consists of two branches. One performs a simple max-pooling operation. The other contains 4 wide activation convolutional layers followed by a down-sampling 2D convolution with stride 2. The feature maps of each branch are summed. An up-sampling wide-activation block also consists of two branches. The first consists of a single upsampling layer, which expands the input feature map depth from 32 to 128, applies a wide-activation convolution, and applies the depth-to-space upsampling operation. The other branch concatenates the input feature maps with those from encoder’s skip-connection. These feature maps are then passed through 4 wide activation layers and an upsampling layer. The upsampled feature maps from both branches are then added together.

The final layer of the decoder branch is a 1x1 convolution operation that predicts the pixel values of the output image. In total, the full CNN model consists of 96 convolutional layers with 2.3 million trainable parameters.

S2.3. Training and applying the CNN

To train such a large CNN on consumer GPUs, we used several tricks to reduce memory consumption. First, instead of using batch normalization to stabilize training, we used weight normalization. This enabled us to use smaller training batches (10 patches per batch) without training becoming slow or erratic. Second, we used gradient checkpointing to reduce GPU memory usage at the expense of longer computation time. We trained the network using the Adam stochastic optimizer for 100 epochs of 500 batches each. We initialize the learning rate at 1E-4 and decrement it by ¼ every 25 epochs.

Once trained, image paths and CTF parameters are read in from a STAR file. Each micrograph is then denoised, beginning with the same preprocessing pipeline used to generate training data. Instead of dividing the image into patches, the whole downsampled, phase-flipped image is passed through the trained CNN. Then, the image is padded with zeros in Fourier space and and a soft low-pass filter is applied at the Nyquist frequency of the down-sampled image. When inverse Fourier transformed, the final denoised image has the same pixel size and dimensions as the noisy input image and has the CTF corrected by phase-flipping.

For the 20S proteasome dataset analyzed in this study, we trained the CNN using 843 full-sized even/odd-frame image sums. Because the denoising objective is not particularly sensitive to overfitting, we trained on the same images we then denoised and analyzed (we do not produce a train/test split). Though we used the whole available dataset here, we find that several hundred even/odd pairs are usually sufficient for training effective denoising CNNs. Because modern cryo-EM datasets can have thousands of images, it may be desirable to train the CNN a subset of 500-1000 even/odd sum pairs, since the restore program generates a down-sampled HDF file containing patches of the original images.

Given a micrograph STAR file with dose-weighted image sums (DW) and paired even- and odd-framed sums (EVN,ODD) corrected by MotionCor2, we used the following restore command to train the CNN and generate the denoised images: train.py -m mics_DW_subset.star -s DW,EVN,ODD -r 3.4 -f training_data_noPP.hdf -x model_maxres3p4_noPP --dont_phaseflip denoise.py -m mics_DW.star -r 3.4 -s denoised_maxres3p4_noPP -p trained_CNN_parameters.pkl --dont_phaseflip --dont_flip_phases_back

Here, '-r 3.4' refers to the spatial frequency band-limit (in angstroms) used to Fourier crop the training data and '--dont_phaseflip' determines how the CTF is applied. Other modifiable parameters such as the learning rate and the number of training epochs were left at reasonable default values, which we recommend for other users.

S2.4. Merging denoised and noisy images

Merging with noisy image and the denoised image is performed by high-pass filtering the noisy image and low-pass filtering the denoised image with complementary soft-edged Fourier filters. Instead of Fourier padding into an array of zeros, the denoised image is effectively Fourier padded with the high-frequency information from the original noisy image. If not properly normalized, sharp changes in amplitude can occur in the band of spatial frequencies where the highpass and lowpass filters overlap (the merge band). To avoid this, the amplitudes of the denoised image are scaled by a normalization factor that keeps the standard deviation of amplitudes in the merge band the same after merging the denoised image into the noisy one. The frequency of the lowpass filter and the spectral width of the soft edge (both in angstroms) are parameters the user can specify.

MRC I/O operations use the library *mrcfile*. STAR file/CTF operations use our library pyem. All other image preprocessing is done using numpy, particularly the real FFT/IFFT routines. All CNN operations were performed with the library keras using the Tensorflow backend.

S2.5. Measuring the angular errors between pairs of orientation parameters

Ignoring the in-plan x-y positions of particles, we measured the angular distance between orientations of each denoised and raw particles determined by separate refinements by using a quaternion representation for the rotation (explicitly, the inverse cosine of the norm of the quaternion inner product). The D7 symmetry of the T20S proteasome was taken into consideration when calculating these angular differences. For all quaternion-based calculations, we used the *geom* module of the library *pyem* (Asarnow *et al.*, 2019).

S3. Estimating signal, noise, and bias in denoised cryo-EM images

Once we have denoised cryo-EM images with a trained CNN, we would like to evaluate the SNR of the denoised image and the magnitude of any systematic errors added by the denoiser. Additionally, we would like to estimate these quantities as a function of spatial frequency.

SNR is defined as the ratio of the signal variance and the noise variance (Bershad & Rockmore, 1974, Frank & Alali, 1975):

eq.8 $$SNR = var(S)/var(N) \approx CC/(1 - CC)$$

Here, var(S) and var (N) are variance of signal and noise, and CC are cross correlation between two images of identical object. Correlation-based SNR estimators require a pair of images taken from an identical object under the identical optic conditions. Historically, most practitioners estimated SSNR of cryo-EM image from the Fourier power spectrum, where the background noise spectrum is estimated by fitting a smooth function through the CTF zeros (Booth *et al.*, 2004). This makes the assumption that the oscillating Thon rings in the power spectrum result exclusively from the signal, whereas noise is unaffected by the CTF. Given the power spectrum and background noise spectrum, the SSNR can be estimated as ((P(s) – N(s))/ N(s) (Booth *et al.*, 2004). While it is straightforward to calculate the SSNR from the Fourier power spectrum in this way, it is impossible to estimate or to remove the influence of the bias introduced by the denoising procedure, as bias may also be modulated by the CTF.

However, modern cryo-EM images are collected with high-speed direct electron detectors as stacks of frames. Assuming that the noise is independent in each frame (as we would expect for shot noise), it is possible to estimate the SNR and SSNR using the classic two-image cross-correlation approach (eq. 8) by calculating two sums of odd and even frames.

We assume that a denoised image (D) is the sum of the signal (S), the remaining uncorrected noise (N_d), and some false signal (B) that results from systematic error in the denoiser (the bias of the estimator). We distinguish N_d from the noise in the original image, which we refer to as N_n.

eq.9 $$D = S + N_d + B$$

Thus, we need to estimate var(S), var(N_d), and var(N_n). We also would like to estimate var(B) for the denoised images. We recall several elementary identities of the variance and covariance:

eq.10 $$\mathrm{var}(X) = \mathrm{cov}(X,X)$$

eq.11 $$\mathrm{cov}(X,Y) = E([X-E(X)][Y-E(Y)]) = E(XY) - E(X)E(Y)$$

eq.12 $$\mathrm{cov}(U+V, Y+Z) = \mathrm{cov}(U,Y) + \mathrm{cov}(V,Y) + \mathrm{cov}(U,Z) + \mathrm{cov}(V,Z)$$

eq.13 $$\mathrm{var}(X+Y) = \mathrm{var}(X) + \mathrm{var}(Y) + 2\mathrm{cov}(X,Y)$$

eq.13 is implied by eq.10 and. eq12. If X and Y are statistically independent, E(XY)=E(X)E(Y) and eq.11 implies that cov(X,Y)=0.

Computationally, the covariance of two images can be estimated with:

eq.14 $$\mathrm{cov}(X,Y) = N^{-1} \Sigma_i (X_i-\bar{X})(Y_i-\bar{Y})$$

Here, N is the number of pixels in images X and Y, X_i is the intensity of the ith pixel of image X, and $\bar{X}$ is the mean pixel intensity.

We denote the noisy even and odd image sums as M_1 and M_2 and their denoised versions as D_1 and D_2. From the previous equations, it is straightforward to show with arithmetic that:

eq.15 $$\mathrm{var}(S) = \mathrm{cov}(M_1,M_2)$$

eq.16 $$\mathrm{var}(N_n) = [\mathrm{cov}(M_1,M_1)\mathrm{cov}(M_2,M_2)]^{½} - \mathrm{cov}(M_1,M_2)$$

eq.17 $$\mathrm{var}(N_d) = [\mathrm{cov}(D_1,D_1)\mathrm{cov}(D_2,D_2)]^{½} - \mathrm{cov}(D_1,D_2)$$

eq.18 $$\mathrm{var}(B) = \mathrm{cov}(D_1,D_2) + [\mathrm{cov}(M_1,M_1)\mathrm{cov}(M_2,M_2)]^{½} - 2\mathrm{cov}(M_1,D_2)$$

In eq.16, the first term on the right-hand side is var(S+N), estimated as the geometric mean of $\mathrm{var}(M_1)$ and $\mathrm{var}(M_2)$. Similarly, the first term on the right-hand side of eq.16 is var(S+N+B) and is estimated as the geometric mean of $\mathrm{var}(D_1)$ and $\mathrm{var}(D_2)$. From these equations, we can estimate the SNR of the images before and after denoising:

eq.19 $$\mathrm{SNR\_noisy} = \mathrm{var}(S) / \mathrm{var}(N_n)$$

eq.20 $$\mathrm{SNR\_denoised} = \mathrm{var}(S) / \mathrm{var}(N_d)$$

Intuitively, eq.19 is identical to the expression for the SNR of noisy image pairs used by the Frank and Alali (Frank & Alali, 1975) and originally suggested by Bershad and Rockmore (Bershad & Rockmore, 1974):

eq.21 $cc(M_1,M_2) = cov(M_1,M_2)/[var(M_1)var(M_2)]^{½}$

eq.22 $SNR\_noisy = cc(M_1,M_2) / [1 - cc(M_1,M_2)]$

We can also estimate other potentially interesting quantities such as the ratio of the signal variance and the bias variance (signal-to-bias ratio, SBR).

eq.23 $SBR = var(S)/var(B)$

Finally, we can estimate each of these quantities as a function of spatial frequency. To estimate the covariance of X and Y at some spatial frequency k, we calculate:

eq.24 $cov(X(k), Y(k)) = N_k^{-1} \Sigma_i X(k)_i Y(k)_i*$

where X(k) is the ring of Fourier components in the Fourier transform of X with spatial frequency k, N^k is the number of Fourier components in ring k, Y(k)* denotes the complex conjugate of Y(k), and the sum runs over each corresponding pair of Fourier components, i. This approach treats each ring in the Fourier transforms of X and Y as an independent random variable.
